# Satellite DNA in *Populus* and Molecular Karyotyping of *Populus xiaohei* and Its Derived Double Haploids

**DOI:** 10.3390/plants14193046

**Published:** 2025-10-01

**Authors:** Bo Liu, Xinyu Wang, Wenjie Shen, Meng Wang, Guanzheng Qu, Quanwen Dou

**Affiliations:** 1 Key Laboratory of Adaptation and Evolution of Plateau Biota, Northwest Institute of Plateau Biology, Chinese Academy of Sciences, Xining 810008, China; liubo176@mails.ucas.ac.cn (B.L.); shenwenjie20@mails.ucas.ac.cn (W.S.); 2 Qinghai Provincial Key Laboratory of Crop Molecular Breeding, Northwest Institute of Plateau Biology, Chinese Academy of Sciences, Xining 810008, China; 3 State Key Laboratory of Tree Genetics and Breeding, Northeast Forestry University, Harbin 150040, China; wangxinyu990927@163.com (X.W.); wangmengnefu@163.com (M.W.); gzqu@nefu.edu.cn (G.Q.); 4 College of Life Sciences, University of Chinese Academy of Sciences, Beijing 100049, China

**Keywords:** *P. xiaohei*, doubled haploid, satDNA, molecular karyotype, chromosome variation

## Abstract

Karyotype analysis and the investigation of chromosomal variations in *Populus* are challenging due to its small and morphologically similar chromosomes. Despite its utility in chromosome identification and karyotype evolutionary research, satellite DNA (satDNA) remains underutilized in *Populus*. In the present study, 12 satDNAs were identified from *P. trichocarpa*, and the copy numbers and chromosomal distributions of each satDNA were analyzed bioinformatically in the reference genomes of *P. trichocarpa*, *P. simonii*, and *P. nigra*. Ten satDNA probes for fluorescence in situ hybridization (FISH) were successfully developed and validated on chromosomes of *P. xiaohei* (poplar hybrid *P. simonii* × *P. nigra*). By integrating bioinformatic genomic satDNA distribution patterns with experimental FISH signals, we constructed a molecular karyotype of *P. xiaohei.* Comparative analysis revealed errors in current poplar genome assemblies. Comparative karyotype analysis of *P. xiaohei* and its doubled haploid (DH) lines revealed chromosomal variations in the DH lines relative to the donor tree. The results demonstrate that the newly developed satDNA probes constitute robust cytogenetic tools for detecting structural variations in *Populus*, while molecular karyotyping provides new insights into the genetic mechanisms underlying chromosome variations in *P. xiaohei* and the DH plants derived.

## 1. Introduction

*Populus xiaohei* T. S. Hwang & Liang (2*n* = 2*x* = 38) is an artificial hybrid of *P. simonii* Carr (2*n* = 2*x* = 38) and *P. nigra* L. (2*n* = 2*x* = 38) with maternal *P. simonii* belonging to the Tacamahaca section and paternal *P. nigra* belonging to the Aigeiros section [1]. As a fast-growing poplar species, *P. xiaohei* exhibits excellent resistance to cold, drought, pests, and diseases and is widely distributed in the northern region of the Yellow River Basin in China [2]. Despite the significant adaptability and growth advantages of hybrid poplars, their high heterozygosity renders breeding and basic research challenging. Haploid breeding technology can rapidly produce homozygous lines, thereby significantly reducing the breeding cycle [3]. Haploid and doubled haploid (DH) plants also have unique genetic characteristics, such as high genetic variation rates and broad variation ranges, which offer distinct advantages in molecular biological research and breeding applications [4]. However, haploid breeding in forest trees still faces challenges, such as difficulties in haploid induction and chaotic ploidy levels in regenerated plants. A DH population was successfully developed from the anthers of *P. xiaohei* in previous studies, and several superior homozygous lines hold promise as model plants for use in genetic and breeding research [5].

Karyotype analysis describes the number and appearance of chromosomes in eukaryotes and provides fundamental information about a species. Fluorescence in situ hybridization (FISH) is a powerful tool used in cytogenetic research to determine the abundance and distribution of various types of DNA within chromosomes [6]. Satellite DNA (satDNA) consists of highly amplified tandem repeats, usually located in the heterochromatin regions of chromosomes, such as the centromere and telomere regions, accounting for 0.1–36% of plant genomes [7]. As the fastest-evolving component of the genome, satDNA often exhibits significant differences in sequence, copy number, and chromosomal distribution within species and between closely related species [7,8], which makes satDNA species- or genus-specific, and in some cases, chromosome-specific [9]. The use of satDNA probes has achieved widespread success in chromosomal and subgenomic identification within or among species within a genus, karyotype evolutionary analysis, and the reconstruction of species differentiation patterns [10,11,12,13]. RepeatExplorer2 is a novel version of a computational pipeline that uses graph-based clustering of next-generation sequencing (NGS) reads for de novo repeat identification in a single species and the development of satDNA probes for cytogenetic experiments [14].

The chromosomes of *Populus* (2*n* = 2*x* = 38) are composed of a pair of large chromosomes and 18 pairs of small chromosomes, which are similar at the morphological level, and it is challenging to distinguish most chromosomes using traditional cytogenetic methods [15]. In previous studies, 45S rDNA, 5S rDNA, and telomere sequences have been used as FISH probes for the karyotype analysis of poplar trees; however, only four pairs of chromosomes could be distinguished [16]. With the rapid development of cytogenetic technology, Xin et al. developed a set of universal chromosome painting probes for *Populus* based on the genome of *P. trichocarpa* [15]. They demonstrated a remarkably conserved poplar karyotype based on individually identified chromosomes, and no inter-chromosomal structural rearrangements were observed in *Populus*. We previously developed an effective tandem repeat probe (pPD284_XHY) to study the molecular karyotype of *P. xiaohei* and its DH plants [17]. The combination of pPD284_XHY, 45S and 5S rDNA distinguished six pairs of chromosomes, and three pairs of chromosomes in *P. xiaohei* showed significant differences in hybridization between homologous chromosomes. Comparison of distinguishable chromosomes between *P. xiaohei* and the DH poplar showed that three pairs in the DH exhibited hybridization patterns distinct from those of *P. xiaohei* [17]. Therefore, intrachromosomal structural variations, such as deletions and duplications, may exist within poplar species and DH plants of *P. xiaohei*, and inversions cannot be excluded. These possibilities must be verified using a more diverse set of FISH probes or whole-genome sequencing. However, the availability of chromosomal markers in the genus *Populus* remains very limited, which restricts the analysis of karyotype evolution and the study of the genetic mechanisms of chromosomal variation between and within species.

In this study, we used NGS data of *P. trichocarpa* to identify its satDNA sequences and compared the abundance and chromosomal distribution of satDNA across the reference genomes of *P. trichocarpa*, *P. nigra*, and *P. simonii* using BLASTN [18]. A set of satDNA probes for *Populus* was developed, and molecular karyotyping, chromosome variation, and genetic analyses of *P. xiaohei* and the derived DH lines were performed. This study elucidated the cytogenetic characteristics of *P. xiaohei* and its DH, thereby advancing our understanding of the genetic mechanisms underlying their chromosomal variations. Furthermore, the satDNA probes developed in this study demonstrated significant potential for comparative genomic analyses within the *Populus* genus.

## 2. Results

### 2.1. Identification and Distribution Analysis of SatDNA

RepeatExplorer2 reconstructed eight high-confidence and four low-confidence satDNAs with satellite-typical circular graph topology and the presence of tandemly arranged monomers in contigs (Figure S1). Among them, the probabilities of high-confidence satDNAs were 0.755–0.925, whereas those of low-confidence satDNAs were 0.0638–0.4290. The consensus monomer length of these satDNAs ranged from 66 to 364 bp, with their proportions in the genome ranging from 0.028% to 0.750% (Table S1).

Individual alignment of each monomeric consensus sequence to the *P. trichocarpa*, *P. nigra*, and *P. simonii* genomes was performed to quantify the abundance and distribution of candidate satDNAs in the poplar genome. The results demonstrated substantial interspecific variations in the copy numbers of the 12 satDNAs across the three reference genomes. *P. nigra* exhibited the highest abundance, with 37,846 copies, all of which could be mapped to chromosome-scale pseudomolecules. *P. trichocarpa* displayed intermediate copy numbers (6984), of which 6088 (87.17%) were localized to chromosome-scale pseudomolecules. *P. simonii* contained the fewest copies (2479), with less than half (1118) being mapped to chromosome-scale pseudomolecules. Furthermore, the abundance and chromosomal distribution of the different satDNAs varied in different chromosomes within and among species (Tables S2–S6 and Figures S2 and S3).

Notably, satDNA CL16 encompassed almost all the locations of CL5 and exhibited a broader distribution range in the reference genomes of the three *Populus* species (Figures S2 and S3). Additionally, CL5 and CL16 showed 96% and 85% sequence similarity, respectively, with the repeat sequence pPD284_XHY developed in *P. xiaohei* (Table S1). These findings suggested that the three sequences are homologous. Therefore, we designed probes for only CL16 and did not design probes for CL5 to enhance detection efficiency. Thus, 11 satDNAs were used for probe design and labeling for further applications (Table 1).

### 2.2. Karyotyping of P. xiaohei and Reference Genomes Limitations

**Figure 1:**
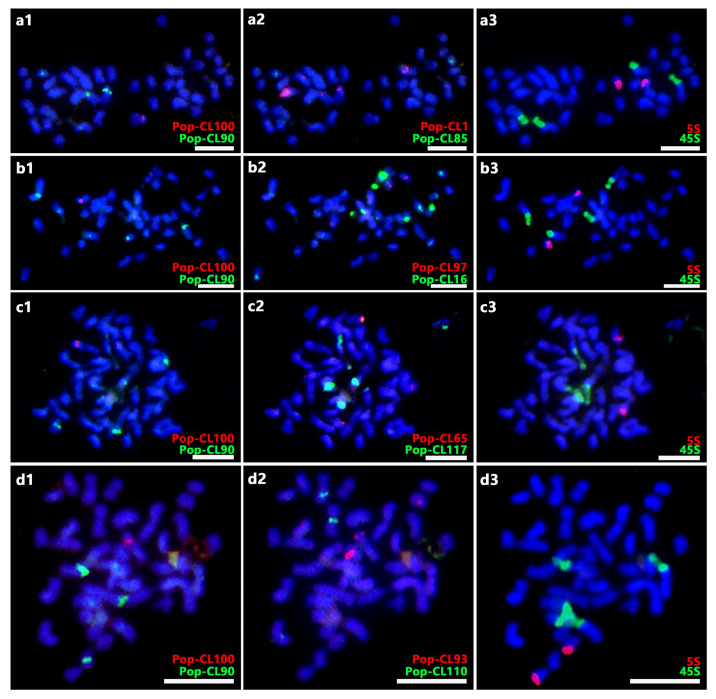
Combination FISH of Pop-CL90 and Pop-CL100 with the remaining probes on mitotic metaphase chromosomes of *P. xiaohei*. Three sequential FISH rounds (1, 2, 3) were carried out on each of four slides (**a**–**d**) to localize the positions of Pop-CL90 and Pop-CL100 on the chromosomes of *P. xiaohei*. Bars = 5 μm.

**Figure 2:**
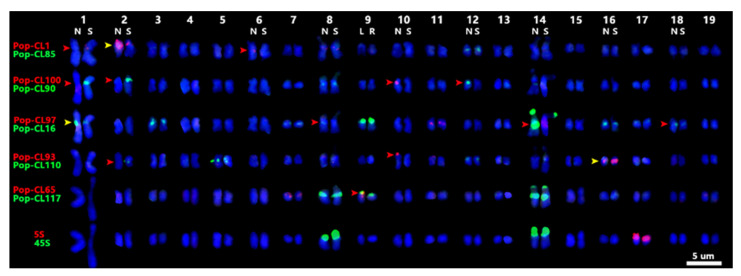
Molecular karyotype of *P. xiaohei*. Red arrows: FISH signal presence/absence; yellow arrows: signal intensity differences. Heterozygous chromosomes were designated: N/S (from *P. nigra* or *P. simonii*), or L/R (unassigned).

FISH was performed with each satDNA probe combined with all the remaining probes on the mitotic metaphase chromosomes of *P. xiaohei* to evaluate the potential of satDNAs as cytogenetic landmarks and to investigate the molecular karyotype of *P. xiaohei*. Ten of the 11 satDNA probes generated detectable signals on the chromosomes of *P. xiaohei* (Figure 1), whereas Pop-CL109 did not produce any detectable signals. These 10 effective satDNA probes, along with 45S rDNA and 5S rDNA, generated a total of 60 FISH signals on 27 chromosomes of *P. xiaohei*. Each of the 27 chromosomes contained one to six FISH signals (Figure 2). Consistent with previous reports, 45S and 5S rDNA were detected as two pairs and one pair of signals, respectively [17]. The FISH signals of the satDNA probes were predominantly localized to the centromeric and telomeric regions, which is consistent with expectations from the genome analysis. Except for Pop-CL85, Pop-CL97, and Pop-CL117 probes, the other seven satDNA probes showed significant signal differences between homologous chromosomes in *P. xiaohei* (Figure 2 and Figure S4).

Based on the comparative information of FISH signals, the abundance and distribution of the identified satDNAs in the parental reference genome of *P. xiaohei*, as well as chromosome length and morphological similarities, 15 of the 19 pairs of chromosomes were distinguished and numbered unambiguously. No signals were detected on chromosomes 4, 13, 15, and 19 in *P. xiaohei* despite the presence of satDNA copies in parental reference pseudomolecules. Among the 15 pairs of chromosomes with FISH signals, the FISH patterns of chromosomes 3, 5, 7, 11, and 17 were homozygous, whereas the remaining 10 were heterozygous (Figure 2). As a direct hybrid of *P. nigra* and *P. simonii*, the karyotype of *P. xiaohei* is expected to combine both parental genomes. Even without analyzing parental karyotypes, the origins of 9 out of 10 heterozygous chromosomes were strongly inferred from FISH signals, guided by the distribution and abundance of satDNAs in the parental reference genomes. The origin of the heterozygous chromosome 9 remained unresolved due to the absence of satDNA CL65 in both parental genomes (Figure 2, Table S3).

**Figure 3:**
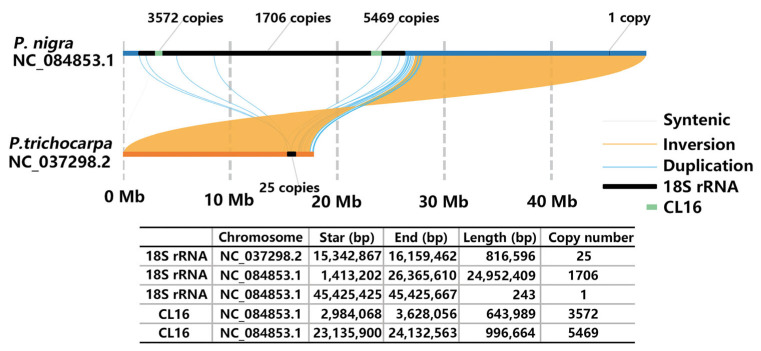
Synteny and distribution of 18S rRNA and satDNA CL16 on pseudomolecule 14 between *P. trichocarpa* and *P. nigra*.

A critical discrepancy exists for Pop-CL16 on chromosome 14N. FISH signals are localized at the end of the short arm and the centromeric region, whereas genomic data indicated positions at 24.7–25.7 Mb (middle) and 45.2–45.9 Mb (terminus) on pseudomolecule 14 of *P. nigra* (Figure 2 and Table S5). This discrepancy arose because the *P. nigra* pseudomolecule 14 (NC_084865.1) used for BLASTN was inverted to maintain synteny with *P. trichocarpa*. Re-orientation of the inverted *P. nigra* pseudomolecule 14 placed 18S rRNA at the sequence start as reported [15,19], resolving the positional discordance of satDNA CL16 (Figure 3). Consequently, the current orientation of *P. trichocarpa* pseudomolecule 14 requires inversion. Furthermore, the scarcity of 18S rRNA units across three poplar reference genomes indicates limitations in rDNA assembly (Tables S2 and S3). This finding is consistent with the results reported for *P. simonii*, highlighting ongoing challenges in rDNA assembly [19].

### 2.3. Chromosome Variation Analysis of DH Materials of P. xiaohei

**Figure 4:**
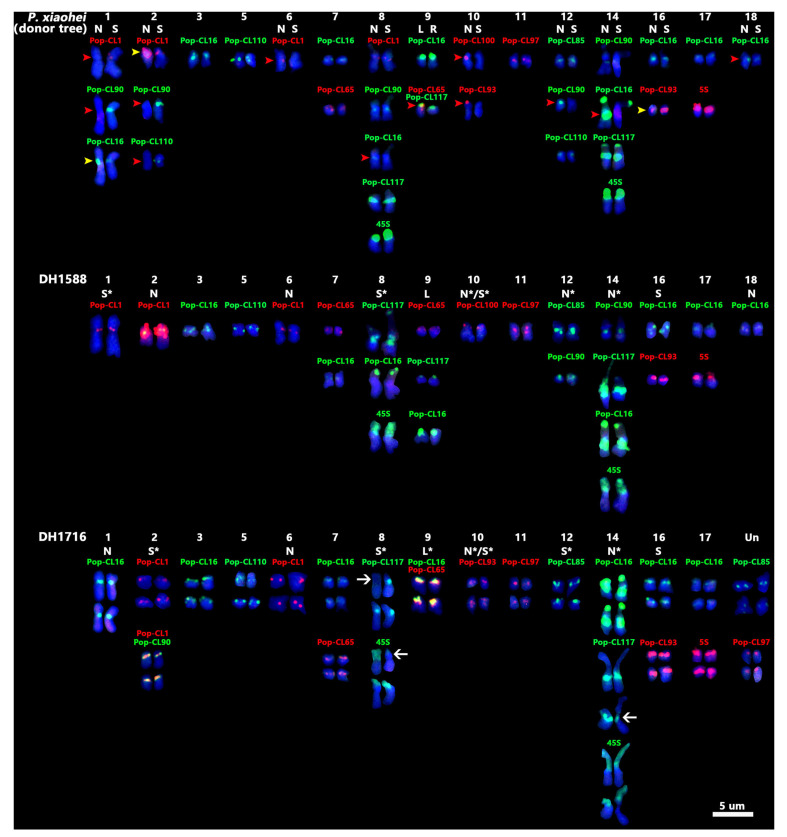
Molecular karyotype comparative analysis of the donor tree (*P. xiaohei*) and the DH materials. The FISH probes are indicated above the chromosomes. Chromosomes 4, 13, 15, and 19 of the DH materials and chromosome 18 of DH1716 are not displayed due to the absence of detectable signals. The meanings of the red and yellow arrows and “N, S, L, R” in the chromosomes of *P. xiaohei* are the same as in Figure 2. The “*” indicates distinct FISH patterns compared to the syntenic chromosomes of *P. xiaohei*. White arrow: heterogeneous signals between homologous chromosomes.

Karyotype analysis was performed on DH materials DH1588 and DH1716 using 10 effective satDNA, 45S rDNA, and 5S rDNA probes to investigate chromosomal variations in anther-derived DH plants of the donor tree (*P. xiaohei*). In total, 38 and 76 chromosomes were detected in DH materials DH1588 and DH1716, respectively (Figures S5 and S6). Stable signals were detected for all probes in both materials, except that no signals were detected for Pop-CL100 in DH1716. The chromosomes of DH1588 and DH1716 were classified into two identical sets and four sets based on the FISH signals, respectively (Figure 4), leading to the conclusion that DH1588 is diploid and DH1716 is tetraploid. This suggested that one and two rounds of chromosome doubling occurred during anther culture or callus regeneration for DH1588 and DH1716, respectively. Similar to the donor tree, FISH signals were detected on all chromosomes of DH materials DH1588 and DH1716, except for chromosomes 4, 13, 15, and 19 in both lines, and chromosome 18 in DH1716 (Figure 4).

Comparative karyotyping of the DH lines with *P. xiaohei* revealed novel chromosomal variations in 5 groups in DH1588 and 6 in DH1716, characterized by donor-divergent FISH patterns, including signal absence, intensity changes, and inter-homologous heterogeneity (Figure S7). DH lines are expected to be homozygous. In this study, DH1716 demonstrated signal discrepancies in the heterochromatic regions between homologous chromosomes 8 and 14 (Figure 4 and Figure S7).

**Figure 5:**
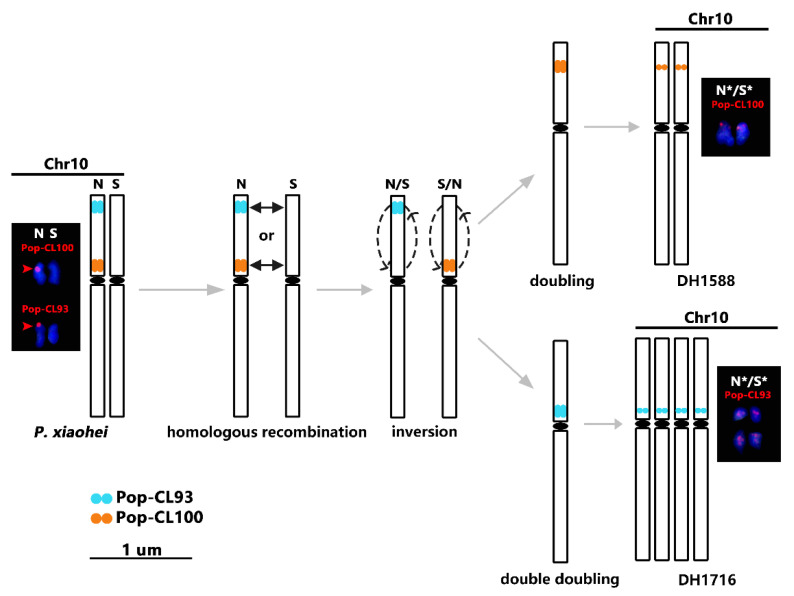
Diagram of potential chromosomal structural variation in the DH lines. The meanings of the red arrows and “*” in the chromosomes are the same as in Figure 4.

FISH signal redistributions in DH lines suggested potential chromosomal structural variations beyond repetitive sequence alterations. In the pseudomolecule 10 of parental genomes of *P. xiaohei*, satDNA CL100 was only distributed within the 5.01–5.98 Mb region, corresponding to centromeric signals on chromosome 10N of *P. xiaohei*. For chromosome 10 of DH1588, only a weak signal for Pop-CL100 was detected near the end of the short arm. This indicates that satDNA CL100 has been redistributed through meiotic crossover between the short arms of chromosome 10 in *P. xiaohei*, followed by a paracentric inversion (Figure 5). Strikingly, DH1716 exhibited a reciprocal configuration: complete loss of Pop-CL100 on the short arm, coupled with emergent centromeric Pop-CL93 signals (Figure 5). These observations further evidence potential chromosomal structural variations on chromosome 10. This suggests that somaclonal variation can be further explored and utilized in DH lines.

## 3. Discussion

SatDNA constitutes 0.1–36% of the plant genome and represents one of the most dynamic genomic components [20,21]. The accumulation and structure of satDNA frequently exhibit sequence and copy number variations among species [8]. In this study, comparative analysis revealed substantial interspecific divergence in satDNA abundance and chromosomal mappability among three poplar genomes, where *P. nigra* demonstrated superior metrics relative to *P. trichocarpa* and *P. simonii*. A similar proportion of satDNA reads (1.3% in *P. trichocarpa*, 2.1% in *P. nigra*, and 0.8% in *P. simonii*) was revealed using RepeatExplorer clustering [22]. Moreover, the variation in satDNA sequence abundance between *P. nigra* and *P. simonii* was reaffirmed by the polymorphic FISH signals revealed in 10 of 19 homologous chromosomes in the interspecific hybrid *P. xiaohei*. Phylogenomic analyses resolve that *P. simonii* and *P. nigra* are more closely related and diverged more recently than *P. trichocarpa* [23]. This divergence paradox suggested that these satDNAs may have undergone copy number variation after the divergence of these two species [7].

Cytogenetic studies of *Populus* are limited. Previous studies have primarily used ribosomal DNA and telomere repeat sequence probes. With the development and combination of cytogenetic and bioinformatics technologies, Xin et al. developed a complete set of 19 chromosome painting probes of *Populus*, localizing 45S rDNA to distal regions of the short arms of chromosomes 8 and 14 in most poplar species, while 5S rDNA was consistently mapped to chromosome 17 across all five studied species [15]. In this study, *P. xiaohei* was found to carry two 45S rDNA loci and one 5S rDNA locus, and we infer that these loci are located at the distal regions of the short arms of chromosomes 8, 14, and 17, respectively. This is consistent with the results of chromosome-specific painting of pachytene chromosomes in *P. simonii* [19]. Comparative karyotypic analyses of *Eucalyptus*, *Nothofagus*, and *Populus* have indicated greater karyotypic stability and conserved chromosome synteny in woody plants than in herbaceous plants [15,24,25]. However, satDNAs are the fastest-evolving components of the genome and may represent chromosomal variations within and between poplar species. In a previous study, we uncovered significant differences in satDNA hybridization signals between homologous chromosomes 1, 8, and 14 of *P. xiaohei* [17]. In the present study, 7 of the 10 effective satDNA probes detected distinct signal differences between homologous chromosomes in *P. xiaohei*. In addition to the previously identified polymorphisms on chromosomes 1, 8, and 14, novel polymorphisms were detected on chromosomes 2, 6, 9, 10, 12, 16, and 18. High differentiation of satDNA distribution between the parental species (*P. nigra* and *P. simonii*) may underlie the heterozygosity observed in *P. xiaohei*. When the 54 FISH signals produced by the 10 satDNA probes in *P. xiaohei* were compared with the parental genome data, 14 signals coincided in position, yet their copy numbers in the genome assemblies appear to have been underestimated relative to the FISH signal intensities. Twenty-two FISH signals lacked cognate satDNA copies in the corresponding pseudomolecules of the parental reference genomes, and four signals exhibited positional mismatches (Figure S4, Table S3). These discordances between hybrid FISH patterns and the parental genome data may stem from assembly errors or sequence polymorphisms between the sequenced accessions of *P. nigra* and *P. simonii* and the actual parents of *P. xiaohei* used in this study, or genomic shock after interspecific hybridization between *P. nigra* and *P. simonii*.

With the rapid advancement of sequencing technologies, several *Populus* species have achieved near-telomere-to-telomere complete genome assemblies, but most species still lack detailed and accurate genomic scaffolding [26,27]. Tandem repeat regions remain a major challenge in genome assembly, primarily because of the misassembly of highly similar repetitive sequences, which often results in underestimating true genome sizes [28]. Molecular cytogenetic techniques can be used to identify errors in genome assembly and facilitate genomic scaffolding. Xin et al. used single-copy DNA probes to demonstrate that the start–end orientation of pseudomolecules 2 and 8 in *P. trichocarpa* requires inversion as well as the association of unanchored sequences with sex chromosomes [15]. Zhao et al. identified discrepancies in 45S rDNA copy numbers and centromeric repeat assemblies in the *P. simonii* genome using chromosome-specific painting [19]. In this study, integrated cytogenetic and bioinformatics analyses revealed that 45S rDNA sequences in the parental reference genomes of *P. xiaohei* exhibited both insufficient and incorrect assemblies. Additionally, multiple satDNA probes detected FISH signals on *P. xiaohei* chromosomes but showed either absence or copy number discrepancies in the corresponding pseudomolecules of *P. simonii* and *P. nigra*. These results suggested persistent assembly errors in the 45S rDNA and satDNA regions of *P. nigra* and *P. simonii*. Further advanced sequencing technology, such as T2T sequencing assisted by molecular cytological methods, could be used in the future to improve the integrity and accuracy of the reference genomes of *P. simonii* and *P. nigra*.

DH materials have substantial value in genetic improvement and fundamental biological studies of *Populus*. Ninety-six DH plant lines have been established through anther culture using *P. xiaohei* as the donor tree [5]. The DH materials used in this study were directly derived from *P. xiaohei*, whose homologous chromosomes are the recombinant types of *P. xiaohei*. Different hybridization patterns in the three chromosomes of the DH poplar were detected by comparison with the donor tree [17]. This study revealed that additional chromosomes with different hybridization patterns deviated markedly from the presumed recombinant types. Chromosome 10 may have undergone chromosomal structural variations in both DH materials. Phenotypic and genotypic changes have been documented many times in plants regenerated through tissue culture and are often termed somaclonal variations [29]. DH materials derived from anther cultures are highly susceptible to somaclonal variations, particularly to chromosomal structural variations [29,30,31]. The expected DH content is homozygous. However, in this study, DH1716 demonstrated signal discrepancies in the heterochromatic regions between homologous chromosomes 8 and 14, manifesting as the absence or reduction of 45S rDNA and Pop-CL117 signals. Compared with euchromatin, heterochromatic regions generally replicate later in the cell cycle, rendering their integrity more susceptible to cell cycle fluctuations [32]. Late-replicating heterochromatin, combined with nucleotide pool imbalances, may induce chromosomal rearrangements during tissue culture [33]. This suggested that karyotypic divergence between homologous chromosomes within DH materials may result from repeat sequence elimination, amplification, or chromosomal rearrangements during tissue culture. This implies that somaclonal variation generated by chromosomal variation can be further explored and utilized in DH lines.

Our previous study demonstrated that chromosome 8 in DH and one of the homologous chromosomes in *P. xiaohei* share highly similar FISH patterns [17]. In DH1588 and DH1716, the FISH signals on five and three chromosomes were consistent with one of the corresponding syntenic chromosomes in *P. xiaohei*, respectively. Although the five chromosomes in DH1588 and the six chromosomes in DH1716 exhibited different FISH patterns from those of the donor tree, most of the signals were consistent with one of the corresponding syntenic chromosomes of *P. xiaohei*. The most obvious chromosome was chromosome 14. Both DH materials retained a strong signal of Pop-CL16 in the centromeric regions, a diagnostic feature unique to chromosome 14N of *P. xiaohei*. This suggested that intact chromosomes or large linkage disequilibrium segments could be transferred from the hybrid and DH material progenies.

## 4. Materials and Methods

### 4.1. Plant Materials

Branches of *P. xiaohei* (approximately 30 years old) were collected from the campus of Northeast Forestry University, China, and DH plants (DH1588 and DH1716) were directly derived from the anther culture of *P. xiaohei* plants (donor tree) using the method described by Liu et al. [5].

### 4.2. satDNA Identification and Genome Distribution Analysis

*P. trichocarpa* next-generation sequencing data (SRR13324523) was obtained from the National Center for Biotechnology Information (NCBI) database. After quality control, one million reads were analyzed using RepeatExplorer2 (Laboratory of Molecular Cytogenetics, Institute of Plant Molecular Biology, Biology Centre, Czech Academy of Sciences, České Budějovice, Czech Republic) to identify satDNAs [14]. Reference genomes of *P. trichocarpa* (GCF_000002775.5), *P. nigra* (GCF_951802175.1), and *P. simonii* (GCA_007826005.2) were subjected to collinearity analysis using JCVI [34]. Prior to this, pseudomolecules 2 and 8 of *P. trichocarpa* were inverted as Xin et al. reported [15]. Based on collinearity with *P. trichocarpa*, we re-ordered and determined the start–end orientation of the pseudomolecules for *P. nigra* and *P. simonii* (Figure S8). BLASTN was used to identify potential satDNA units within the adjusted genomes of *P. trichocarpa*, *P. nigra* and *P. simonii* [18]. Distribution heatmaps of satDNA in the three poplar genomes were created using TBtools-II (v2.327) [35], visualizing the alignment results. The sequence similarity of potential satDNAs and tandem repeats of pPD284_XHY, which were previously identified [17], was estimated using BLASTN [18].

### 4.3. Probe Design and Probe Labeling

Oligonucleotide probes for FISH experiments were designed based on the consensus sequences of satDNAs to characterize the chromosomal distribution of satDNA families. The probes were synthesized by Sangon Biotech (Shanghai) Co., Ltd. (Shanghai, China). The 5S (4 oligonucleotides, TAMRA-labeled) and 45S rDNA (12 oligonucleotides, FAM-labeled) multiplex probes (24–31 nt), designed from conserved angiosperm sequences [36], Sangon Biotech (Shanghai) Co., Ltd. (Shanghai, China).

### 4.4. Chromosome Preparation

Vigorously growing branches of the plant were inserted into the rooting medium. After rooting, the secondary root tips (approximately 1 cm long) were cut and placed in a water-filled polyethylene tube immersed in a mixture of ice and water for 24 h. After the pretreatment, the root tips were transferred to a polyethylene tube fixed in 3:1 (*v*/*v*) ethanol/glacial acetic acid. Subsequently, the root tips were transferred to 70% alcohol and stored at −20 °C in a refrigerator for long-term preservation. Chromosomal spreading and slide preparation were performed as previously described [17].

### 4.5. Fluorescent In Situ Hybridization

FISH experiments were performed as described by Liu et al. with minor modifications [17]. The chromosome preparations were denatured in 0.2 M NaOH in 70% ethanol for 10 min at room temperature; subsequently, they were rinsed in cold 70% ethanol (−20 °C) for 1 h and quickly air-dried. The amount of hybridization mixture for each slide was the same as that previously reported [17]. Hybridization was performed overnight at 37 °C. Sequential FISH with three rounds of hybridization of the same mitotic spread of *P. xiaohei* was performed. The first two rounds of hybridization were performed using different satDNA probes, and the third round of hybridization was conducted using 5S and 45S rDNA as anchors. Each satDNA probe was co-hybridized with all other satDNA probes to precisely determine the relative positions of the designed satDNAs on the chromosomes of *P. xiaohei*. After the first round of FISH, at least ten cells with clear signals were captured. Subsequently, the slides were heated at 55 °C for 5 min and washed three times in 2×SSC at 42 °C for 5 min each to remove the FISH signals. Next, we proceeded to the next round of hybridization with different probes.

Chromosomes were stained with 4′,6-diamidino-2-phenylindole (DAPI) in a VectaShield antifade solution. The photomicrographs were obtained using an Olympus BX-63 fluorescence microscope equipped with a DP-80 CCD device. Each chromosome in three complete mitotic metaphase cells of *P. xiaohei* was measured (Table S7).

## 5. Conclusions

Twelve satDNA sequences were identified from *P. trichocarpa*, and ten effective FISH probes applicable to *Populus* were developed and identified. The molecular karyotype of *P. xiaohei* was analyzed, and 15 pairs of chromosomes with FISH signals were identified. The genomic data of *P. nigra* and *P. simonii* showed insufficient and incorrect assembly at the satDNA and 45S rDNA loci. Additionally, the start–end of the current pseudomolecule 14 of *P. trichocarpa* was incorrectly oriented. By comparing the FISH patterns of *P. xiaohei* and its derived DH materials, chromosomal variations potentially caused by crossing over or tissue culture were revealed, and some possible chromosomal structural variations in the DH materials were also inferred. Our work provides the first comprehensive molecular karyotype of *P. xiaohei*; FISH patterns were used to infer parental origin, detect chromosomal changes in the DH lines, and reveal hidden assembly errors in reference genomes of *Populus*. These findings demonstrate the utility of satDNA probes in *Populus* genome research and provide insights into chromosomal variations in DH breeding systems.

## Figures and Tables

**Table 1:** Information on oligonucleotide probes for FISH analysis.

Satellite DNA	Probe Name	Fluorochrome Label	Sequence (5′ to 3′)
CL1	Pop-CL1	TAMRA	TTCGACAGCCCAAACAGCCC
CL16	Pop-CL16	FAM	GATCCGACCGTTGGATCGCGG
CL65	Pop-CL65	TAMRA	ACTCGGTGACGAGGAGGTCT
CL85	Pop-CL85	FAM	TCTGCAGCGACTCAGTTTTCG
CL90	Pop-CL90	FAM	GGTGACGAGTGGATTCGTCA
CL93	Pop-CL93	TAMRA	GGTGACCTGCCCATCTTAAA
CL97	Pop-CL97	TAMRA	CTACTGCTGGCTCAGAAACCG
CL100	Pop-CL100	TAMRA	GATCAGCACTGGCATAGTGT
CL109	Pop-CL109(a)	FAM	TTCCCCAGCTGATGCCTGGTT
	Pop-CL109(b)	FAM	GCACAATCCCTGTTGACTCG
CL110	Pop-CL110	FAM	CAACGAAACTGCTTCCACGG
CL117	Pop-CL117	FAM	AAAACAGAAATCCTAATTACT

Note: The SatDNA probe Pop-CL109 comprises two oligonucleotide sequences, designated (a) and (b).

## Data Availability

The original contributions presented in the study are included in the article; further inquiries can be directed to the corresponding author.

## Supplementary Materials

The following supporting information can be downloaded at https://www.mdpi.com/article/10.3390/plants14193046/s1. Figure S1: De novo repeat identification by RepeatExplorer2 read cluster analysis; Figure S2: Distribution of SatDNA sequences on the *P. trichocarpa* genome; Figure S3: Distribution of SatDNA sequences on the *P. nigra* and *P. simonii* genome; Figure S4: The molecular karyotype diagram of *P. xiaohei*; Figure S5: FISH of satDNA probes on mitotic metaphase chromosomes of DH1588; Figure S6: FISH of satDNA probes on mitotic metaphase chromosomes of DH1716; Figure S7: Diagram of chromosomal variation patterns in the DH materials; Figure S8: Genome collinearity analysis of *P. trichocarpa*, *P. nigra* and *P. simonii*. Table S1: The information of putative satellite repeats; Table S2: The copy number of SatDNA in the *P. trichocarpa* reference genome; Table S3: The copy numbers of satDNAs and 18S rDNA in the reference genomes of *P. nigra* and *P. simonii*, and a comparative analysis of SatDNA loci using FISH signals and genome assembly data; Table S4: The alignment results of satDNAs with the *P. trichocarpa* reference genome; Table S5: The aligement results of satDNAs with the *P. nigra* reference genome; Table S6: The aligement results of satDNAs with the *P. simonii* reference genome; Table S7: Chromosome measurements of *P. xiaohei*.

## References

[1] Wang C., Tung S. (1982). New Taxa of *Populus* (II). Bull. Bot. Res..

[2] Du Z.W., Zheng T.C., Li S., Zang L.N., Qu G.Z., You X.L. (2015). Rapid Propagation and Regeneration System of *Populus simonii* × *Populus nigra*. Bull. Bot. Res..

[3] Qu Y., Fernie A.R., Liu J., Yan J. (2024). Doubled haploid technology and synthetic apomixis: Recent advances and applications in future crop breeding. Mol. Plant.

[4] Dwivedi S.L., Britt A.B., Tripathi L., Sharma S., Upadhyaya H.D., Ortiz R. (2015). Haploids: Constraints and opportunities in plant breeding. Biotechnol. Adv..

[5] Liu C., Wang S., Liu Y., Wang M., Fan E., Liu C., Zhang S., Yang C., Wang J., Sederoff H.W. (2023). Exceptionally high genetic variance of the doubled haploid (DH) population of poplar. J. For. Res..

[6] Younis A., Ramzan F., Hwang Y.J., Lim K.B. (2015). FISH and GISH: Molecular cytogenetic tools and their applications in ornamental plants. Plant Cell Rep..

[7] Garrido-Ramos M.A. (2017). Satellite DNA: An Evolving Topic. Genes.

[8] Lower S.S., McGurk M.P., Clark A.G., Barbash D.A. (2018). Satellite DNA evolution: Old ideas, new approaches. Curr. Opin. Genet. Dev..

[9] Klemme S., Banaei-Moghaddam A.M., Macas J., Wicker T., Novák P., Houben A. (2013). High-copy sequences reveal distinct evolution of the rye B chromosome. New Phytol..

[10] Schmidt T., Heitkam T., Liedtke S., Schubert V., Menzel G. (2019). Adding color to a century-old enigma: Multi-color chromosome identification unravels the autotriploid nature of saffron (*Crocus sativus*) as a hybrid of wild *Crocus cartwrightianus* cytotypes. New Phytol..

[11] Wei L., Liu B., Zhang C., Yu Y., Yang X., Dou Q., Dong Q. (2020). Identification and characterization of satellite DNAs in *Poa* L.. Mol. Cytogenet..

[12] He Y., He J., Zhao Y., Zhang S., Rao X., Wang H., Wang Z., Song A., Jiang J., Chen S. (2024). Divergence of 10 satellite repeats in *Artemisia* (*Asteraceae: Anthemideae*) based on sequential fluorescence in situ hybridization analysis: Evidence for species identification and evolution. Chromosome Res..

[13] Heitkam T., Weber B., Walter I., Liedtke S., Ost C., Schmidt T. (2020). Satellite DNA landscapes after allotetraploidization of quinoa (*Chenopodium quinoa*) reveal unique A and B subgenomes. Plant J..

[14] Novák P., Neumann P., Macas J. (2020). Global analysis of repetitive DNA from unassembled sequence reads using RepeatExplorer2. Nat. Protoc..

[15] Xin H., Zhang T., Wu Y., Zhang W., Zhang P., Xi M., Jiang J. (2020). An extraordinarily stable karyotype of the woody *Populus* species revealed by chromosome painting. Plant J..

[16] Hu B., Dong F., Wang C., Qi L., Song W., Chen C. (2012). Multicolor Fluorescence in Situ Hybridization of Seven *Populus* Species-ribosomal DNA and Telomere Repeat Sequence. Acta Sci. Nat. Univ. Nankaiensis.

[17] Liu B., Wang S., Tao X., Liu C., Qu G., Dou Q. (2021). Molecular Karyotyping on *Populus simonii* × *P. nigra* and the Derived Doubled Haploid. Int. J. Mol. Sci..

[18] Camacho C., Coulouris G., Avagyan V., Ma N., Papadopoulos J., Bealer K., Madden T.L. (2009). BLAST+: Architecture and applications. BMC Bioinf..

[19] Zhao Y., Liu G., Wang Z., Ning Y., Ni R., Xi M. (2023). Oligo-FISH of *Populus simonii* Pachytene Chromosomes Improves Karyotyping and Genome Assembly. Int. J. Mol. Sci..

[20] Garrido-Ramos M.A. (2015). Satellite DNA in Plants: More than Just Rubbish. Cytogenet. Genome Res..

[21] Neumann P., Oliveira L., Čížková J., Jang T.-S., Klemme S., Novák P., Stelmach K., Koblížková A., Doležel J., Macas J. (2021). Impact of parasitic lifestyle and different types of centromere organization on chromosome and genome evolution in the plant genus *Cuscuta*. New Phytol..

[22] Usai G., Mascagni F., Natali L., Giordani T., Cavallini A. (2017). Comparative genome-wide analysis of repetitive DNA in the genus *Populus* L.. Tree Genet. Genomes.

[23] Zhang B., Zhu W., Diao S., Wu X., Lu J., Ding C., Su X. (2019). The poplar pangenome provides insights into the evolutionary history of the genus. Commun. Biol..

[24] Ribeiro T., Barrela R.M., Bergès H., Marques C., Loureiro J., Morais-Cecílio L., Paiva J.A. (2016). Advancing *Eucalyptus* Genomics: Cytogenomics Reveals Conservation of *Eucalyptus* Genomes. Front. Plant Sci..

[25] Acosta M.C., Premoli A.C. (2018). Understanding the extensive hybridization in South American *Nothofagus* through karyotype analysis. Bot. J. Linn. Soc..

[26] Liu W., Liu C., Chen S., Wang M., Wang X., Yu Y., Sederoff R.R., Wei H., You X., Qu G. (2024). A nearly gapless, highly contiguous reference genome for a doubled haploid line of *Populus ussuriensis*, enabling advanced genomic studies. For. Res..

[27] Gao W., Wang S., Jiang T., Hu H., Gao R., Zhou M., Wang G. (2025). Chromosome-scale and haplotype-resolved genome assembly of *Populus trichocarpa*. Hortic. Res..

[28] Gao S., Yu H., Wu S., Wang S., Geng J., Luo Y., Hu S. (2018). Advances of sequencing and assembling technologies for complex genomes. Hereditas.

[29] Bairu M.W., Aremu A.O., Van Staden J. (2011). Somaclonal variation in plants: Causes and detection methods. Plant Growth Regul..

[30] Akagbuo N., Jaja E. (2013). A review of somaclonal variation in plantain (*Musa* spp.): Mechanism and applications. J. Appl. Biosci..

[31] Aswathi T.P., Kumari I.P., Rafeekher M., Reshmi C.R., Anuradha T. (2025). Somaclonal Variants in Ornamental Foliage: A Review. J. Adv. Biol. Biotechnol..

[32] Lee M., Phillips R.L. (1988). The Chromosomal Basis of Somaclonal Variation. Annu. Rev. Plant Biol..

[33] Gahan P. (1976). Molecular Aspects of Gene Expression in Plants. Biochem. Soc. Trans..

[34] Tang H., Krishnakumar V., Zeng X., Xu Z., Taranto A., Lomas J.S., Zhang Y., Huang Y., Wang Y., Yim W.C. (2024). JCVI: A versatile toolkit for comparative genomics analysis. iMeta.

[35] Chen C., Wu Y., Li J., Wang X., Zeng Z., Xu J., Liu Y., Feng J., Chen H., He Y. (2023). TBtools-II: A “one for all, all for one” bioinformatics platform for biological big-data mining. Mol. Plant.

[36] Waminal N.E., Pellerin R.J., Kim N.S., Jayakodi M., Park J.Y., Yang T.J., Kim H.H. (2018). Rapid and Efficient FISH using Pre-Labeled Oligomer Probes. Sci. Rep..

