# Supplementary material for: Satellite DNA in Populus and Molecular Karyotyping of Populus xiaohei and Its Derived Double Haploids
Source: Plants (Basel). 2025 Oct 1;14(19):3046. doi: 10.3390/plants14193046 (PMC12525766; doi:10.3390/plants14193046)
Supplement: Supplementary file 1 [file plants-14-03046-s001.zip › Supplemental Figures.pdf]

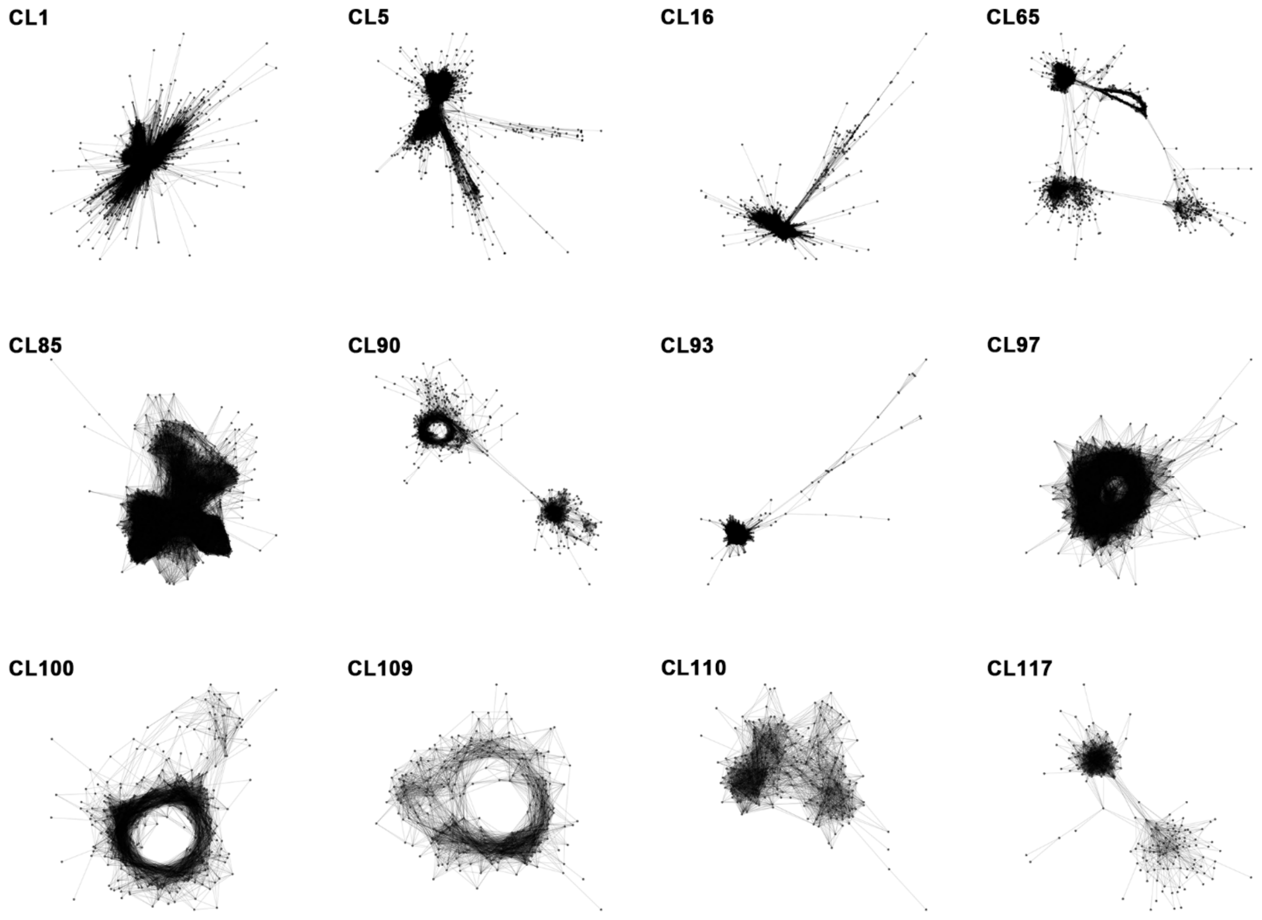

Figure S1. *De novo* repeat identification by RepeatExplorer2 read cluster analysis. Circular graphs represent the SatDNA-typical read clusters. The RepeatExplorer2 cluster number (CL) are indicated.

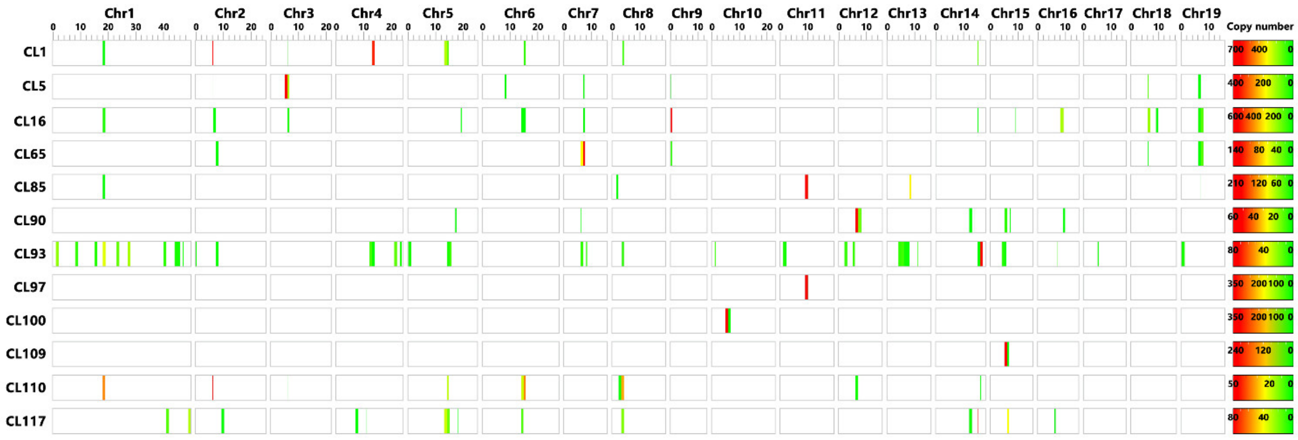

Figure S2. Distribution of SatDNA sequences on the *P. trichocarpa* genome. The genomic distribution of SatDNAwas mapped using BLASTN and visualized using TBtools-II.

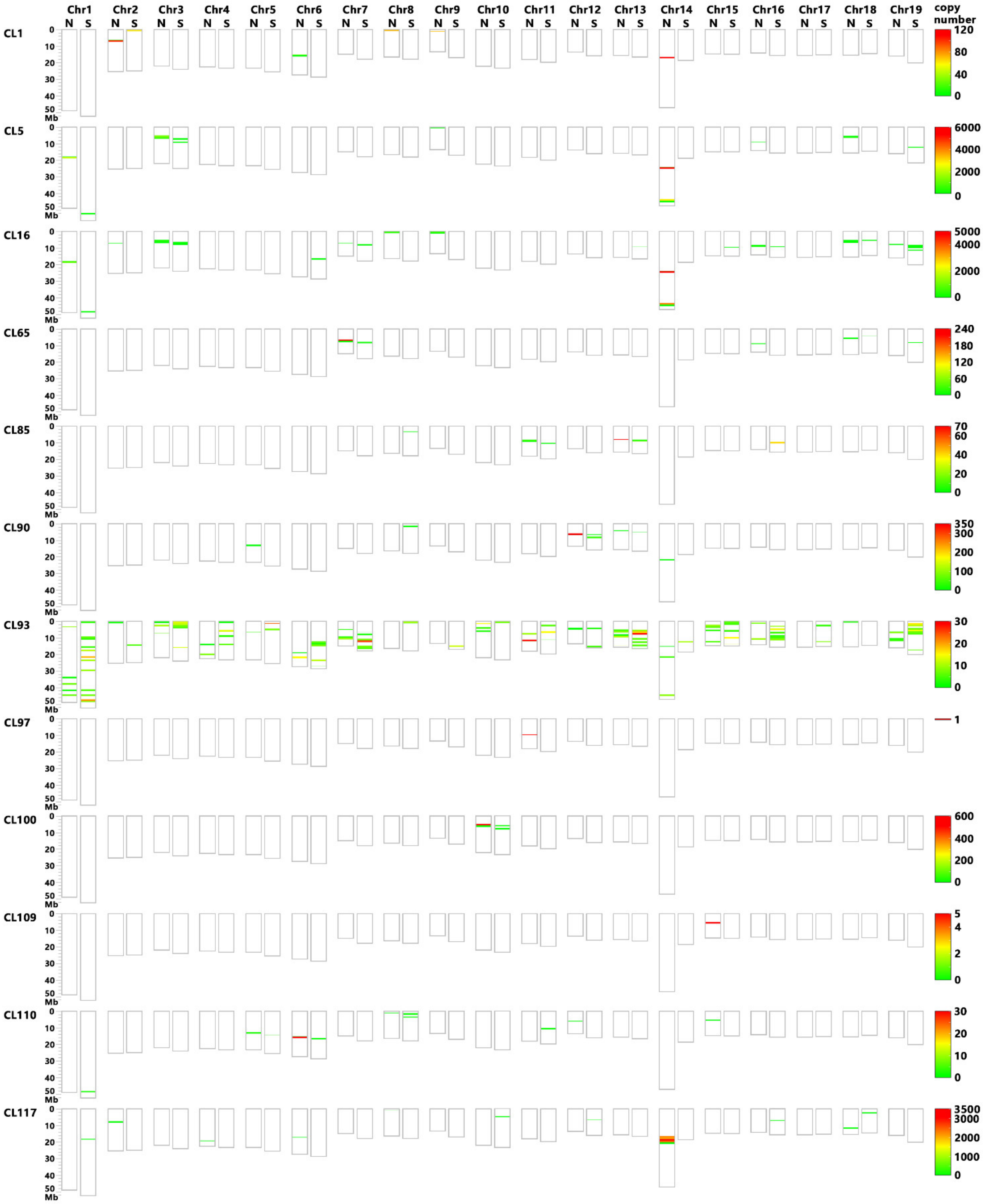

Figure S3. Distribution of SatDNA sequences on the *P. nigra* and *P. simonii* genome. The genomic distribution of SatDNA was mapped using BLASTN and visualized using TBtools-II. The "N" column represents the pattern diagram of *P. nigra*, and the "S" column represents the pattern diagram of *P. simonii*.

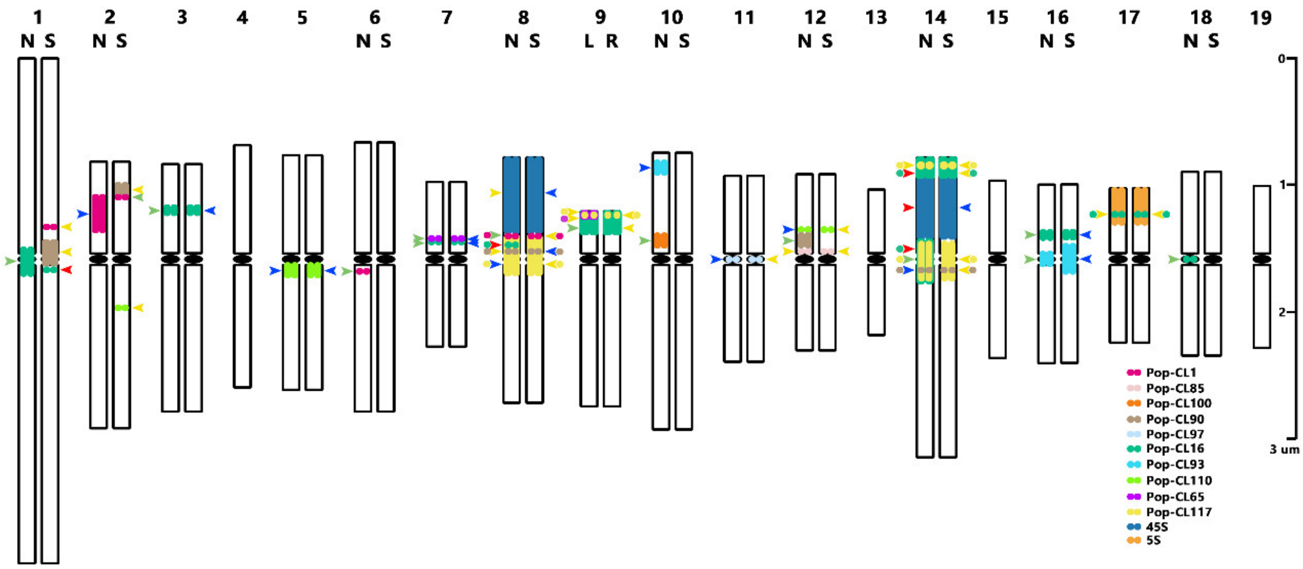

Figure S4. The molecular karyotype diagram of *P. xiaohei*. The parental reference pseudomolecules (from *P. nigra* and *P. simonii*) were used for comparison with FISH signals. Green arrows: FISH signals were consistent with their respective satDNA location and abundance on the corresponding parental reference pseudomolecules. Blue arrows: FISH signals were consistent with their respective satDNA locations but may have lower copy numbers. Yellow arrows: FISH signals lacked corresponding satDNA copies on the corresponding parental reference pseudomolecules. Red arrows: FISH signals showed positional mismatch with their respective satDNA locations. If an arrow points to overlapping FISH signals, the arrow is followed by a circle that matches the color of the signal it indicates. The meanings of "N, S, L, and R" are the same as in Figure 2.

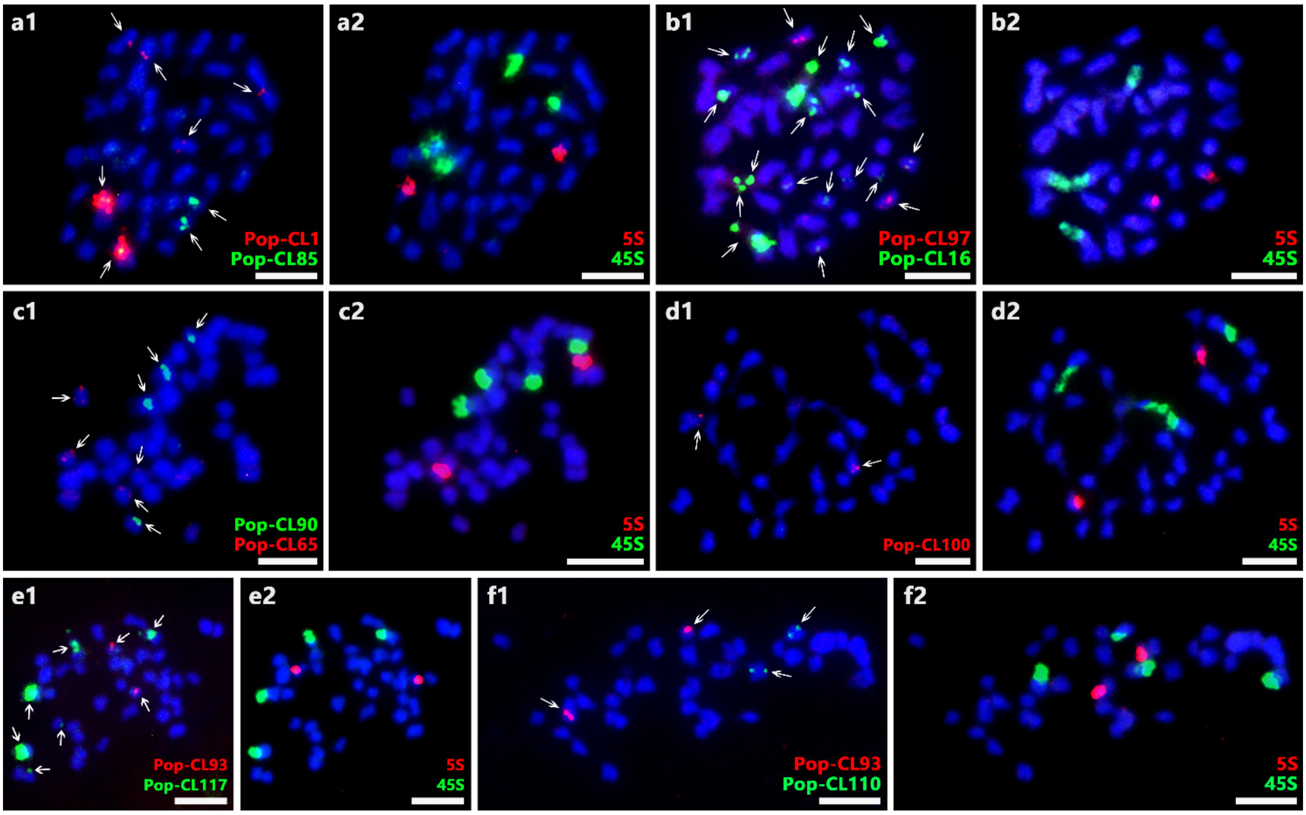

Figure S5. FISH of satDNA probes on mitotic metaphase chromosomes of DH1588. Arrows indicate the positions of FISH signals from satDNA probes. Bars = 10 um.

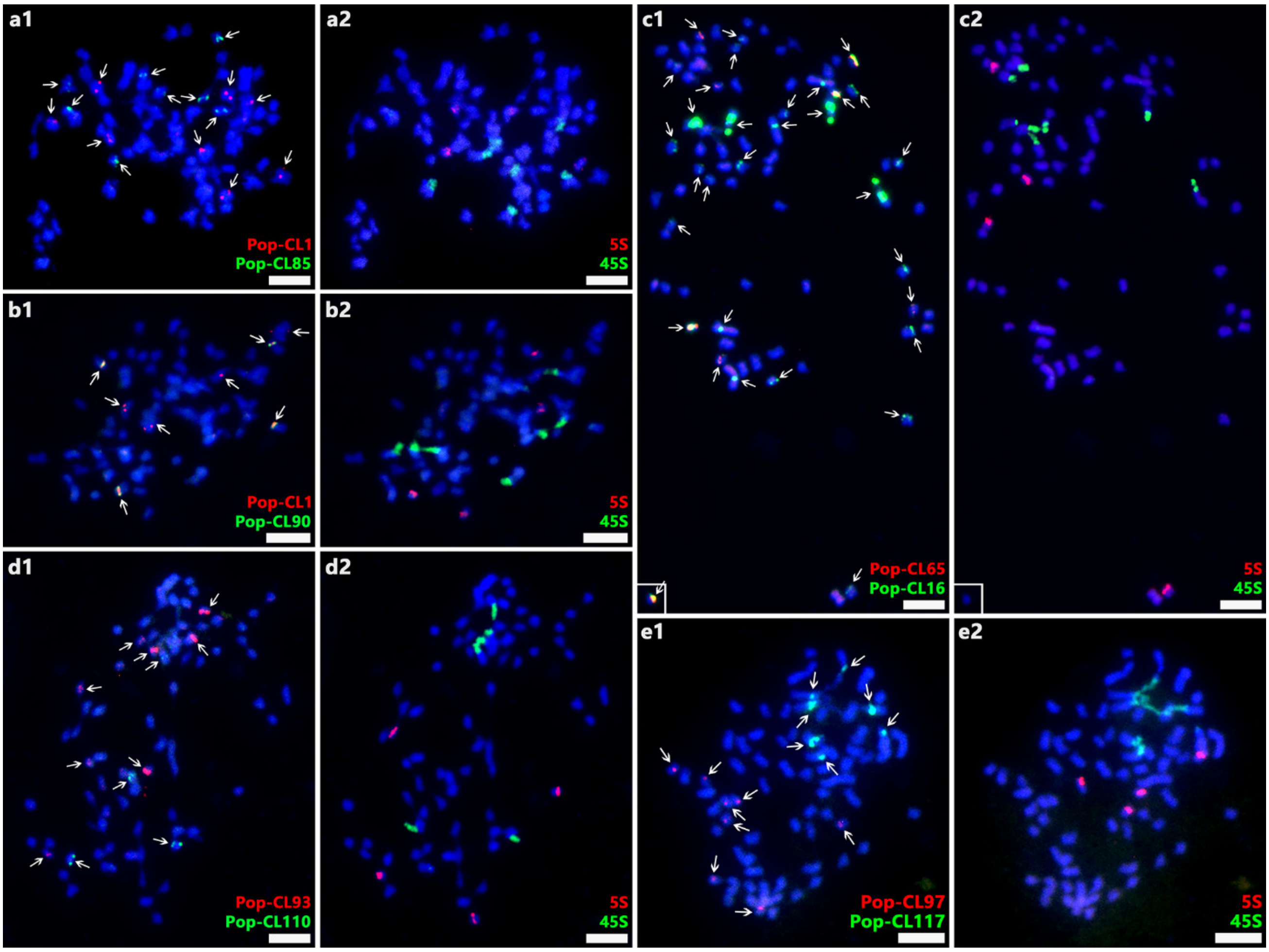

Figure S6. FISH of satDNA probes on mitotic metaphase chromosomes of DH1716. Arrows indicate the positions of FISH signals from satDNA probes. Bars = 10 um.

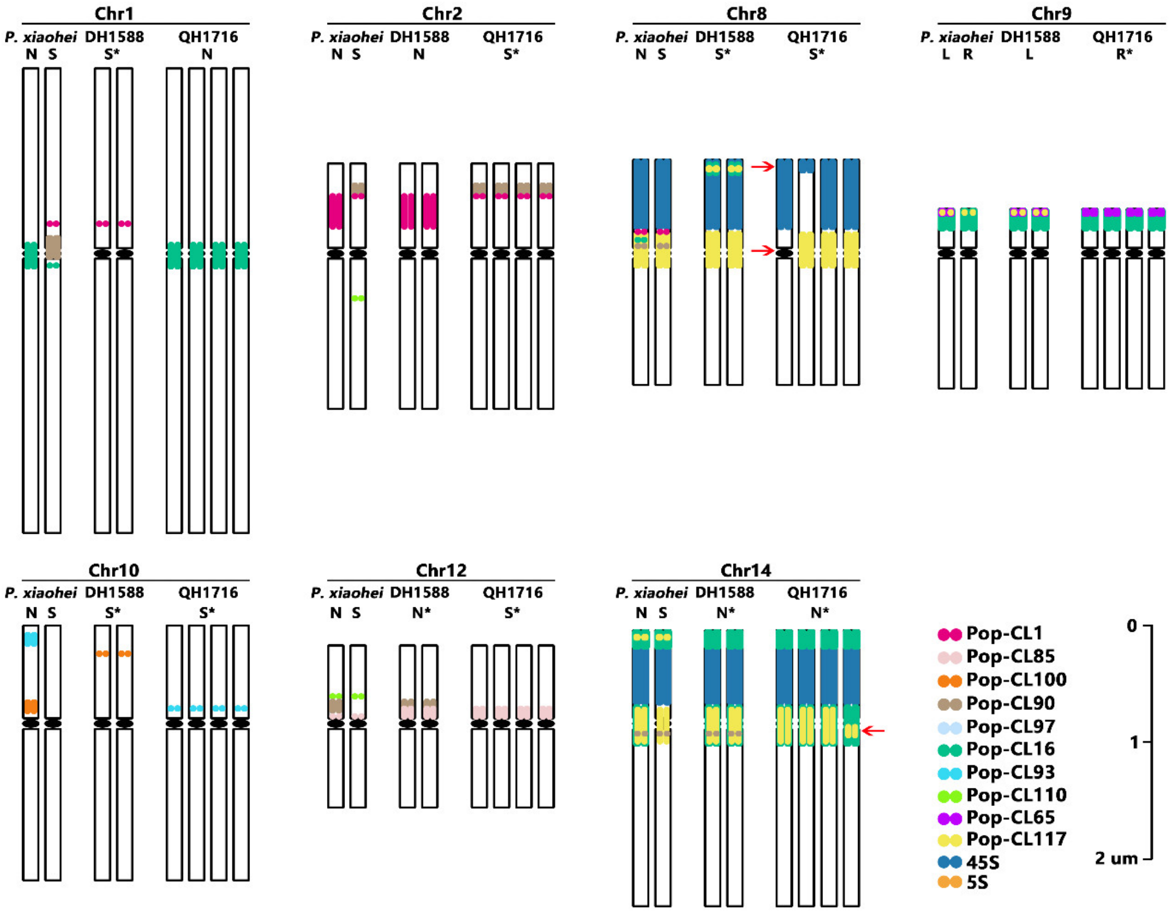

Figure S7. Diagram of chromosomal variation patterns in the DH materials. The meanings of "N, S, L, R and *" are the same as in Figure 4. Red arrow: polymorphic signals among homologous chromosomes of DH1716.

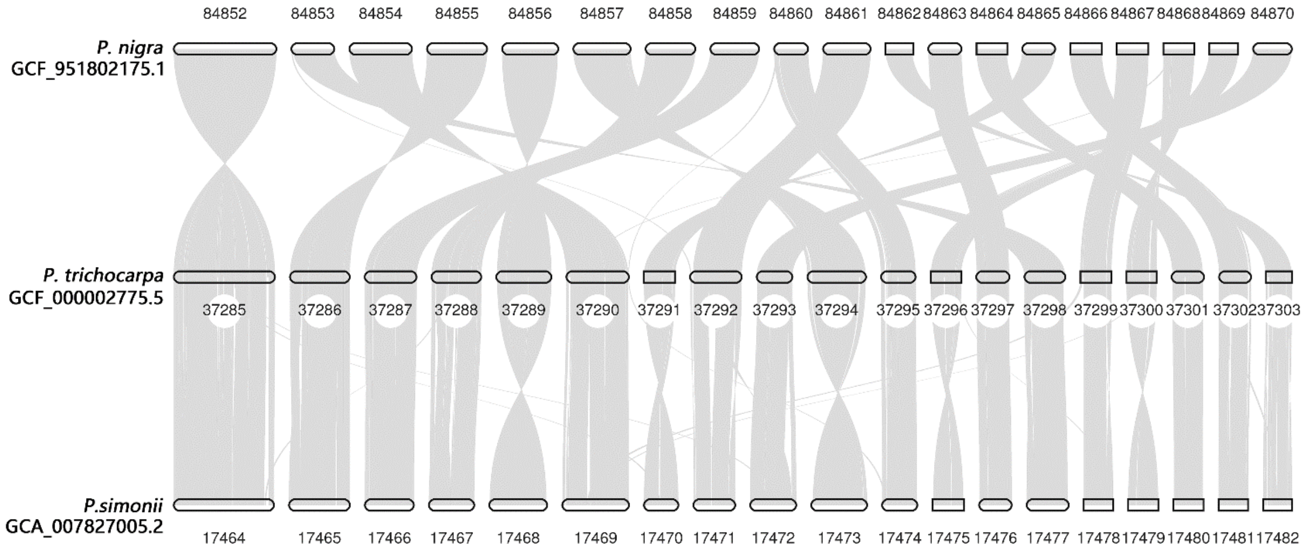

Figure S8. Genome collinearity analysis of *P. trichocarpa*, *P. nigra* and *P. simonii*. Chromosomes 1 to 14 are arranged from left to right. Due to the long full names of the chromosomes in the reference genomes, which are inconvenient to display in the image, only partial numbers (such as "37285") are used to represent the full name (NC_037285.2). Before the collinearity comparison, pseudomolecules 2 and 8 of *P. trichocarpa* were inverted as previously described [1].

References

1. Xin, H.; Zhang, T.; Wu, Y.; Zhang, W.; Zhang, P.; Xi, M.; Jiang, J. An extraordinarily stable karyotype of the woody *Populus* species revealed by chromosome painting. *Plant J.* **2020**, *101*, 253-264. doi:10.1111/tpj.14536.
